# Identifying rice field weeds from unmanned aerial vehicle remote sensing imagery using deep learning

**DOI:** 10.1186/s13007-024-01232-0

**Published:** 2024-07-16

**Authors:** Zhonghui Guo, Dongdong Cai, Yunyi Zhou, Tongyu Xu, Fenghua Yu

**Affiliations:** 1 School of Information and Electrical Engineering, Shenyang Agricultural University, Shenyang, 110866 China; 2 National Digital Agriculture Regional Innovation Center (Northeast), Shenyang, 110866 China; 3 Key Laboratory of Smart Agriculture Technology in Liaoning Province, Shenyang, 110866 China; 4 Key Laboratory of Smart Agriculture in the South China Tropical Region, Ministry of Agriculture and Rural Affairs, Guangzhou, 510640 China

**Keywords:** Rice field weeds, Target detection, Transformer, DETR, UAV

## Abstract

**Background:**

Rice field weed object detection can provide key information on weed species and locations for precise spraying, which is of great significance in actual agricultural production. However, facing the complex and changing real farm environments, traditional object detection methods still have difficulties in identifying small-sized, occluded and densely distributed weed instances. To address these problems, this paper proposes a multi-scale feature enhanced DETR network, named RMS-DETR. By adding multi-scale feature extraction branches on top of DETR, this model fully utilizes the information from different semantic feature layers to improve recognition capability for rice field weeds in real-world scenarios.

**Methods:**

Introducing multi-scale feature layers on the basis of the DETR model, we conduct a differentiated design for different semantic feature layers. The high-level semantic feature layer adopts Transformer structure to extract contextual information between barnyard grass and rice plants. The low-level semantic feature layer uses CNN structure to extract local detail features of barnyard grass. Introducing multi-scale feature layers inevitably leads to increased model computation, thus lowering model inference speed. Therefore, we employ a new type of Pconv (Partial convolution) to replace traditional standard convolutions in the model.

**Results:**

Compared to the original DETR model, our proposed RMS-DETR model achieved an average recognition accuracy improvement of 3.6% and 4.4% on our constructed rice field weeds dataset and the DOTA public dataset, respectively. The average recognition accuracies reached 0.792 and 0.851, respectively. The RMS-DETR model size is 40.8 M with inference time of 0.0081 s. Compared with three classical DETR models (Deformable DETR, Anchor DETR and DAB-DETR), the RMS-DETR model respectively improved average precision by 2.1%, 4.9% and 2.4%.

**Discussion:**

This model is capable of accurately identifying rice field weeds in complex real-world scenarios, thus providing key technical support for precision spraying and management of variable-rate spraying systems.

## Introduction

During the growth period of rice, competition for soil nutrients and water between rice and weeds can lead to the loss of water and fertilizer resources. Additionally, the proliferation of weeds can contribute to the emergence and spread of diseases and pests. Weeds in rice fields have become a critical biological threat limiting rice yield and quality [1]. Therefore, effective weed control is a necessary step to achieve high and stable rice production.

The characteristics of rice field environments, including soft and wet soil, low-lying terrain, and narrow spaces, impose certain limitations on traditional mechanical weed control methods [2]. In this context, unmanned aerial vehicles (UAVs) have demonstrated unique applicability due to their flexible maneuverability. In recent years, with the improvement of payload capacity and performance of agricultural UAVs, aerial spraying has become the mainstream method for weed control in rice fields [3].

Currently, there is a problem of indiscriminate spraying in weed control using agricultural UAVs in rice fields. The widespread spraying may not accurately target weed locations, leading to low pesticide utilization rates and potential negative environmental impacts [4, 5]. Utilizing high-resolution rice field remote sensing images captured by UAVs for precise weed identification and generating variable-rate prescription maps can enable targeted pesticide application based on weed locations and quantities, addressing this issue effectively [6].

Barnyard grass is one of the most common weeds in rice fields, belonging to the same Poaceae family as rice. Both share a high degree of similarity in appearance and growth habits [7]. In rice field images obtained by UAVs, barnyard grass weeds often occupy only a few dozen pixels, representing typical small targets. The recognition process for such small targets is prone to false positives or negatives due to lighting conditions and mutual occlusion. The high similarity between barnyard grass and rice, coupled with the small size of the targets and the complex and dynamic background, poses a significant challenge for accurate identification of barnyard grass in rice fields based on UAV remote sensing images [8].

In recent years, deep learning approaches have demonstrated significant potential in weed identification tasks [9, 10]. Deep learning, with advantages such as end-to-end learning, high-level feature learning, and large-scale data-driven capabilities, has rapidly emerged as the mainstream method in the field of object detection [11]. Deep learning can directly learn end-to-end from large-scale annotated weed image data, automatically extracting visual features required for weed classification without the need for manual feature design and selection. Furthermore, with the expansion of datasets, deep models show continuous improvement in performance and adaptability to different agricultural environments [12].

Modern object detectors have two typical architectures: based on CNNs and based on Transformers. In recent years, extensive research has been conducted on CNN-based object detectors. These detectors are primarily categorized into two-stage networks and one-stage networks, with the representative models being the R-CNN series and the YOLO series [13, 14]. Zhang et al. [15] embedded the CBAM attention mechanism after the pooling layers in the latter part of VGG19, forming the VGG19-CBAM structure as the optimal backbone feature extraction network for the Faster R-CNN model. They utilized this model for weed detection in soybean fields, achieving an average recognition accuracy of 99.16%, with an average recognition speed of 336 ms per image. Gallo et al. [16] collected over 3000 weed remote sensing data using drones in a chicory plantation, creating a weed dataset in chicory plant production. They trained a YOLOv7 model on this dataset for weed target detection, achieving an average recognition accuracy of 56.6%.

The Transformer-based object detector utilizes the self-attention mechanism to generate contextual representations of input sequences, enabling it to effectively capture long-range dependencies within the input sequences. This is particularly crucial for tasks involving complex structures and long sequences [14]. In recent years, Transformer-based detectors have made significant progress in performance, thanks to researchers' relentless efforts in accelerating training convergence and reducing optimization challenges [17]. Zhu et al. [14] pointed out that when Transformer components are initialized, attention modules apply almost identical attention weights to all pixels in the feature map, leading to a longer training time to converge. To address this issue, they proposed a deformable attention module, combining the advantages of deformable convolution sparse spatial sampling and the relationship modeling capability of transformers, to overcome the slow convergence problem in DETR models. Li et al. [18] attributed the slow convergence of DETR models to the instability of bipartite graph matching, resulting in inconsistent optimization objectives in the early training stages. To resolve this issue, they introduced noisy ground truth bounding boxes into the Transformer decoder, effectively reducing the difficulty of bipartite graph matching and accelerating convergence. However, despite achieving a certain degree of improvement in convergence speed and overall performance, the model exhibits poor performance in detecting small targets. Current research has demonstrated that integrating multi-scale feature layers into the model can effectively enhance its detection performance for small targets [19].

In this paper, we propose an RMS-DETR model, which enhances the DETR model's ability to detect small targets by introducing multi-scale feature layers into the DETR framework. Existing research indicates that low-level semantic information typically contains more fine-grained and local features, which may be more distinctive and sensitive for small targets [20–22]. Therefore, we differentially design the various feature layers of multi-scale features. For high-level semantic information, we apply Transformer structures to extract features, fully integrating context information from different perceptual domains. For low-level semantic information, we use a more computationally efficient CNN structure for feature extraction and encoding. Subsequently, effective fusion of the two types of features is achieved through cross-scale feature fusion, leveraging their respective advantages and forming an information-rich feature space. In traditional Transformer structures, due to all heads sharing the same input features and relying on isolated learning with non-shared parameters, there is often a highly homogeneous and redundant representation across different heads [23, 24]. To reduce computational redundancy, we adopt a novel structure called Cascaded Group Attention (CGA) module to replace the traditional Transformer structure. This module provides different channel subsets of features as input for each head, allowing each head to learn more unique features, thereby enhancing the model's learning ability and reducing computational redundancy. The introduction of multi-scale feature layers inevitably increases the model's computational complexity and slows down the inference speed. In this study, we use an efficient and parallelizable Partial convolution (PConv) in the RMS-DETR model to replace conventional convolution, aiming to maximize the model's inference speed.

## Experimental design

### Data collection

**Fig. 1:**
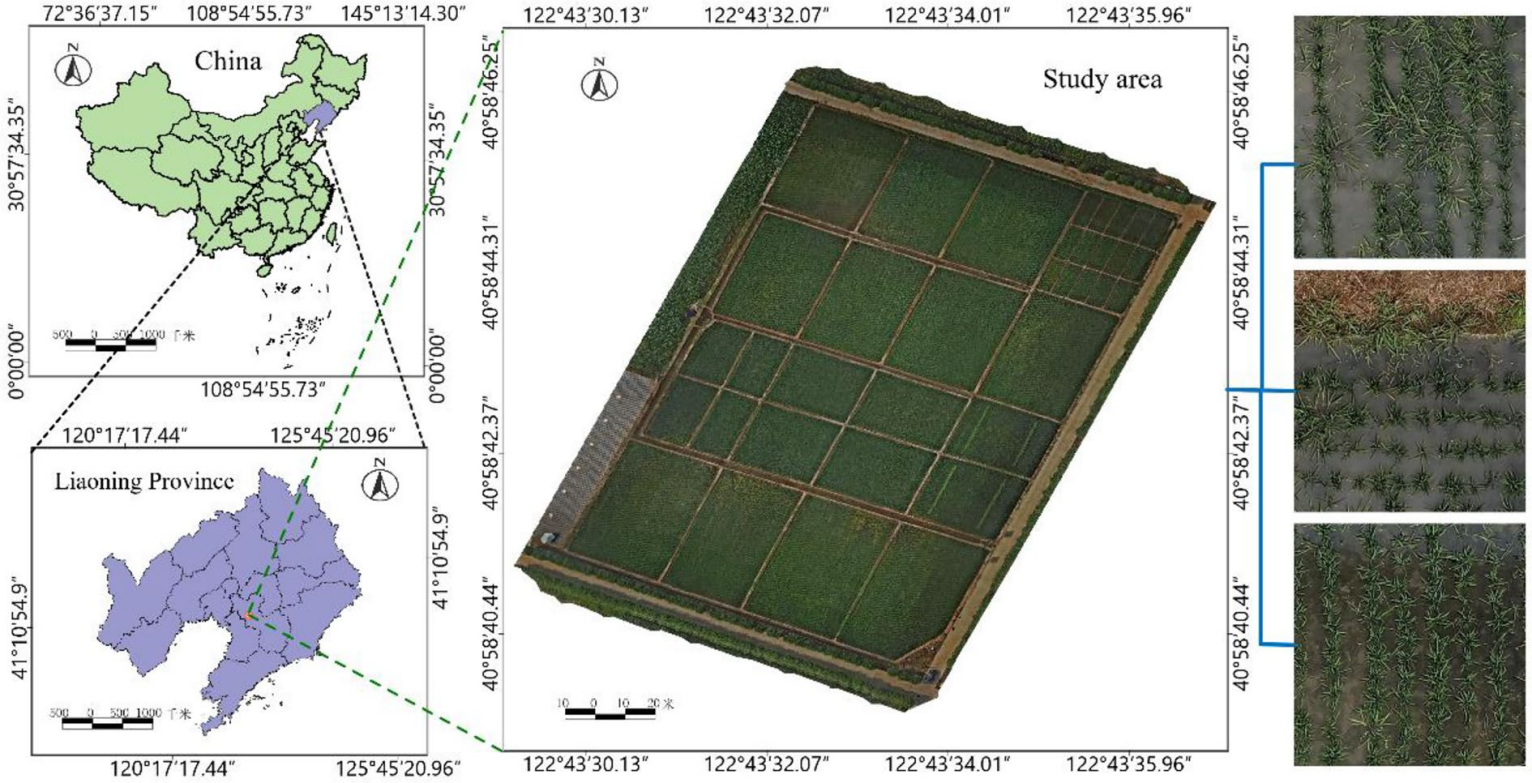
Schematic diagram of the experimental area

In the actual rice production process in the northeastern region of China, farmers adopt a weed control strategy as follows: firstly, before transplanting rice seedlings, they use soil-sealing herbicides in the soil to prevent weed growth. Secondly, during the rice tillering stage, they employ drones to apply herbicides once again for weeding. Finally, after the rice enters the tillering stage, farmers conduct another weeding operation based on the distribution of weeds in the field. After the first two weeding operations, during the rice tillering stage, the weeds in the rice fields are relatively fewer and unevenly distributed, making it more suitable for precise herbicide application. Our research focuses on identifying weeds in rice fields during the tillering stage to support variable-rate precision herbicide spraying using drones. Therefore, between May and June 2022, at the experimental field of Shenyang Agricultural University in Haicheng City, Liaoning Province, UAV were utilized to collect remote sensing data for both rice and barnyard grass during the tillering and panicle initiation stages. The experimental area measured 165 m in length, 97 m in width, with a total area of 16,005 square meters, as illustrated in Fig. 1. The DJI M300 drone served as the flight platform, flying at an altitude of 30 m and equipped with the Zenmuse P1 lens with an effective pixel count of 45 million. The sensor size is 35.9 × 24 mm, with a pixel size of 4.4 μm and aperture of F2.8. The Ground Sampling Distance (GSD) is 0.375 cm/pixel. To ensure image registration accuracy, the drone followed a predetermined flight path with 80% forward overlap and 80% side overlap. Images were captured in a vertical perspective to cover the entire experimental field. The collected image resolution was 8192 × 5460 pixels. DJI Terra (V3.4.4) [25] was used for image registration and fusion of the acquired rice field remote sensing images. To prevent disturbances to both rice plants and weeds caused by strong winds and ensure the accuracy of subsequent image registration and fusion, UAV remote sensing data collection was conducted under weather conditions with wind speeds below level 4, as specified by the GBT28591-2012 standard. A total of 171 UAV remote sensing images of rice fields were collected.

### Dataset generation

**Fig. 2:**
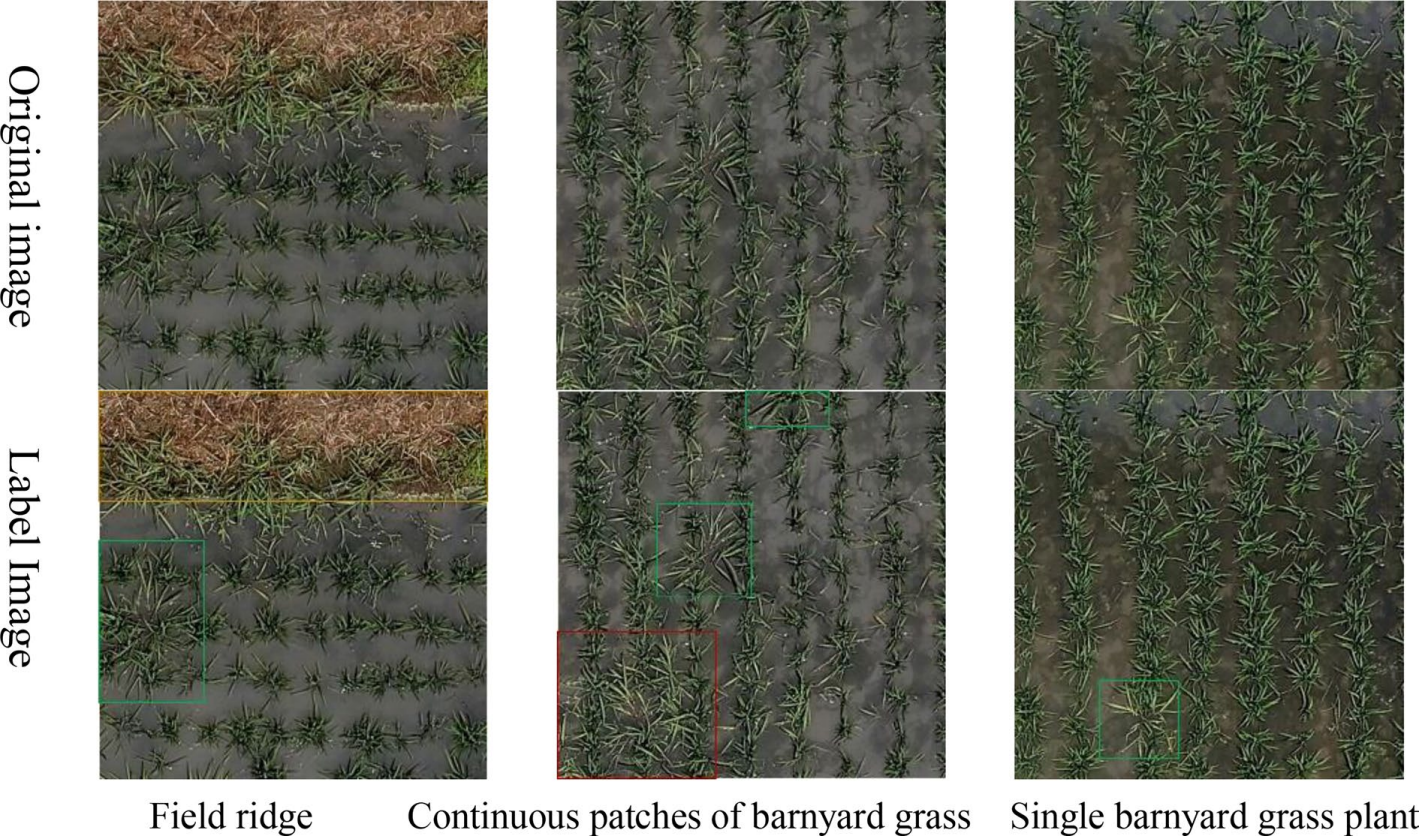
Example of a remote sensing image dataset of weeds in rice fields. The yellow-highlighted boxes in the image indicate the "Field ridge" labels, the green-highlighted boxes represent the "Single barnyard grass plant" labels, and the red-highlighted boxes correspond to the "Continuous patches of barnyard grass" labels

To avoid inconsistencies in annotation when the same target appears in different images, this study initially used DJI Terra to register and fuse the collected unmanned aerial vehicle remote sensing data of weeds. Subsequently, the registered and fused images were segmented into non-overlapping sub-images of 600 × 600 pixels each. After image segmentation, a total of 3,094 rice field weed remote sensing images were obtained. Weed distribution in the field is uneven, with dense areas showing continuous weed growth, while sparse areas exhibit individual weed plants. Therefore, during the manual annotation process, barnyard grass was classified into two types: continuous patches of barnyard grass and single barnyard grass plants. The Labelme software (v4.5.6) [26] was employed for manual annotation. The schematic diagram of the dataset is shown in Fig. 2, and the sample quantities after annotation are presented in Table 1, Based on the definitions of small targets (area ≤ 322), medium targets (322 < area ≤ 962), and large targets (area > 962) from the COCO dataset [27], we calculated the area of each target bounding box. In our constructed rice field weed dataset, the proportions of small, medium, and large targets are 13.7%, 33.6%, and 52.7%, respectively The dataset was partitioned into training, validation, and testing sets at a ratio of 7:2:1, with no duplicate data between sets.

**Table 1:** Number of samples in the dataset

Label category	Numbers of original images	Total number of images after augmentation
Field ridge	438	1752
Continuous patches of barnyard grass	218	872
Single barnyard grass plant	2876	11,504

### Data augmentation

To enhance the model's robustness to the aforementioned samples, we augmented the training dataset by fourfold through techniques such as random cropping, color jittering, noise addition, and random rotation. This resulted in a dataset containing a total of 14,128 images, with the sample distribution outlined in Table 1.

## Materials and methods

### Methodology employed in this study

**Fig. 3:**
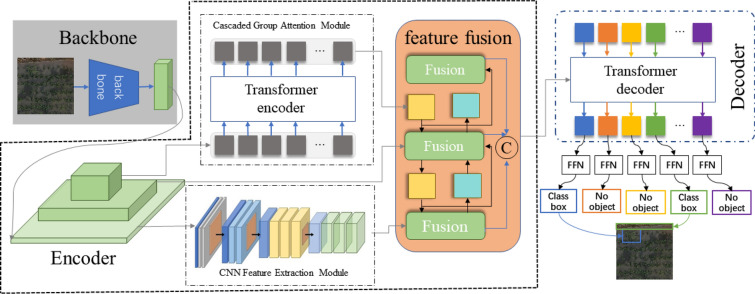
Network architecture

Model Overview: To address the limitations of the DETR model in detecting small objects [28] inspired by the Deformable DETR model, we introduced distinct multiscale feature layers into the DETR model, creating the RMS-DETR model.RMS-DETR. The purpose of this modification is to better adapt to small targets, particularly the detection of single barnyard grass plants in rice fields. The model framework, illustrated in Fig. 3, consists of three core components: Backbone, Encoder, and Decoder. We designed a hybrid encoder, which comprises a feature extraction module and a cross-scale feature fusion module. In the feature extraction module, we differentiated the design for different feature layers. The high-level semantic feature layers employ a Transformer structure to emphasize the extraction of contextual information related to grass and rice, while the low-level semantic feature layers efficiently extract detailed grass features using a CNN structure. The cross-scale feature fusion module effectively combines the features extracted by the Transformer and CNN structures across different scales. This unique design of the hybrid encoder organically combines features from different levels, creating favorable conditions for the overall performance improvement of the model. To better extract grass features in the high-level semantic feature layers, we introduced the CGAmodule, replacing the traditional multi-head attention mechanism to enhance learning ability while reducing computational burden. Last but not least, to further improve the inference speed, RMS-DETR adopts the efficient and parallelizable PConv, successfully replacing conventional convolution operations. The innovative design enhances the performance of our model in small object detection tasks., especially in the detection of grass.

We selected a rice field weed image with dimensions of 600 × 600. Firstly, we preprocessed the image by resizing it to 640 × 640× 3 using bilinear interpolation. The resized image was then fed into the Backbone module for initial feature extraction. In the middle layers of the Backbone, we concurrently inserted multiple convolutional layers to perform multi-scale convolution operations on the feature map, generating fixed-dimensional multi-scale feature representations. This resulted in three multi-scale feature layers labeled as s3, s4, and s5, with dimensions of 80 × 80 × 128, 40 × 40 × 256, and 20 × 20 × 512, respectively. The high-level semantic feature layer s5 was input into the transformer structure for feature encoding, producing the encoded feature layer y1 with dimensions of 20 × 20 × 512. Simultaneously, the low-level semantic feature layer s3 was input into a CNN network for feature extraction, yielding the feature layer y2 with dimensions of 80 × 80 × 256. Finally, we input y1, y2, and s4 into the feature fusion module for cross-scale feature fusion. The output is the fused feature layer y3 with dimensions of 80 × 80 × 512. This y3 serves as our final feature representation, which is then input into the subsequent decoding layers to accomplish weed recognition predictions.

**Fig. 4:**
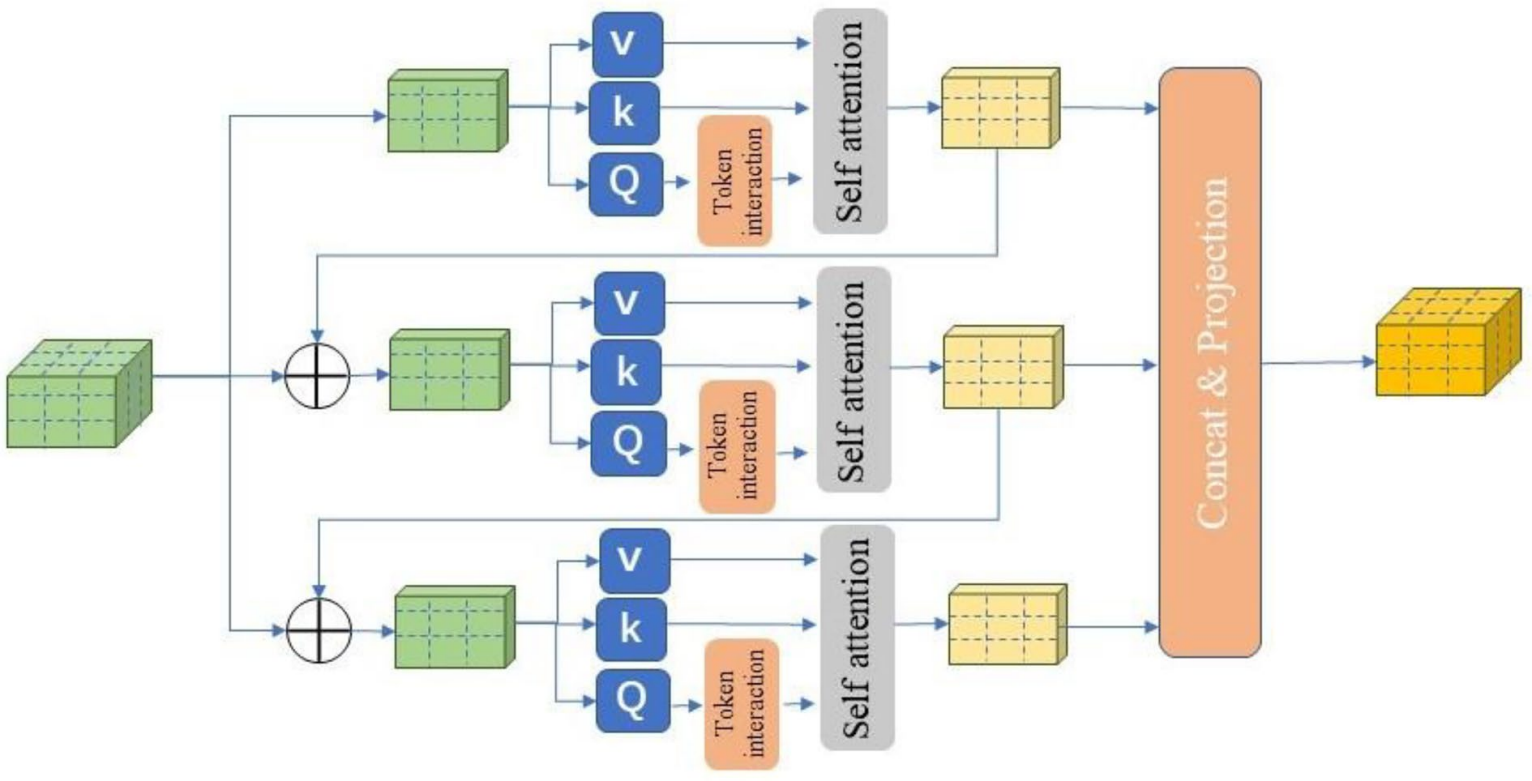
Network structure of the CGAModule

Feature Extraction Based on CGA: In traditional Transformer structures, due to shared input features, different heads may learn redundant information. Simultaneously, because parameters are not shared, each head independently learns weights and feature representations, potentially leading to overly similar features across different heads, lacking diversity. To overcome this issue, this study introduces the CGAmodule. This module provides each head with different channel subsets of features as input, enabling each head to learn more unique features. Additionally, it cascades output features among heads, thereby enhancing the model's learning ability and reducing computational redundancy [29]. As illustrated in Fig. 4, the structure of the CGAmodule can be described as follows:

1 $${\widetilde{X}}_{ij}=Attn({X}_{ij}{W}_{ij}^{Q},{X}_{ij}{W}_{ij}^{K},{X}_{ij}{W}_{ij}^{V})$$

2 $${\widetilde{X}}_{i+1}=Concat{\left[{\widetilde{X}}_{ij}\right]}_{j=1:h}{W}_{i}^{P}$$

where the $j$th head computes the self-attention over ${X}_{ij}$, which is the $j$th split of the input feature ${X}_{i}$, i.e., ${X}_{i}=$ $\left[{X}_{i1},{X}_{i2},\dots ,{X}_{ih}\right]$ and $1\le j\le h$. $h$ is the total number of heads, ${W}_{ij}^{\text{Q}},{W}_{ij}^{\text{K}}$, and ${W}_{ij}^{\text{V}}$ are projection layers mapping the input feature split into different subspaces, and ${W}_{i}^{\text{P}}$ is a linear layer that projects the concatenated output features back to the dimension consistent with the input.

The CGA calculates the attention maps for each head in a cascading manner, adding the output of each head to the subsequent ones. This design encourages the Q, K, V layers to learn feature projections with richer information, progressively improving the capacity of feature representation. Through the cascading structure, this process allows the model to continuously accumulate and propagate richer information in each attention head, contributing to the enhancement of the model's learning ability and further optimizing feature representation:

3 $$X_{ij}^{\prime} = X_{ij} + \tilde{X}_{i(j - 1)} , \quad 1 < j \le h$$

where $X_{ij}^{\prime}$ is the addition of the $j$th input split ${X}_{ij}$ and the $(j-1)$th head output ${\widetilde{X}}_{i(j-1)}$ calculated by Eq. (2). It replaces ${X}_{ij}$ to serve as the new input feature for the $j$th head when calculating the self-attention. Besides, another token.

The CGA can save *h* × FLOPs and parameters since the input and output channels of the QKV layers are reduced by *h* × . Secondly, cascading attention heads can increase the network depth, thereby further enhancing the model capacity without introducing any additional parameters.

**Fig. 5:**
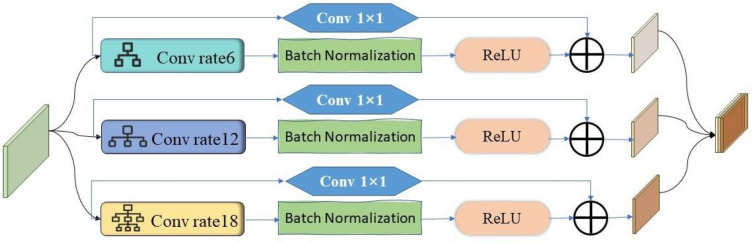
Network structure of the CNN feature extraction module

Feature Extraction Based on CNN Structure: Existing research results indicate that low-level semantic feature layers contain more fine-grained local information, which is crucial and sensitive for the detection of small targets [30]. CNN's convolution and pooling operations aid in extracting local information such as textures and shapes in images, making it easier to capture local features and details in the images. This makes CNN more suitable for extracting and encoding detailed features from low-level semantic feature layers [31, 32].Therefore, in the design of the hybrid encoder for the RMS-DETR model, we utilized a CNN structure in the feature extraction module to extract detailed information about weeds from the low-level semantic feature layer. When using the CNN network to extract low-level details, appropriately expanding the receptive field of the CNN network enables it to capture richer features of the target and surrounding background areas, thereby improving the quality of small target detection [33, 34]. Dilation convolution, compared to regular convolution, can enlarge the receptive field, obtaining broader and richer features, which is crucial for detecting small targets of different scales [35, 36]. Therefore, we employed dilated convolution for feature extraction on the low-level semantic feature layer, as illustrated in Fig. 5.

To capture multi-scale features within different receptive fields, we employed dilated convolutions with dilation factors of 6, 12, and 18 to extract low-level semantic information from the multi-scale feature layers. Here, the kernel size of the dilated convolution is 3 × 3, and dilated convolutions with different dilation factors, along with Batch Normalization and ReLU activation functions, form separate branches. To alleviate potential issues of gradient vanishing or exploding during training, we introduced a residual structure in each branch, including a 1 × 1 convolution layer. The outputs of each branch are obtained by summing them, and these features are then concatenated together. By applying a 1 × 1 convolution operation, we reduced the channel number from 240 to 80, obtaining a globally fused feature representation that incorporates multi-scale contextual information. This helps in capturing subtle features of barnyard grass in the rice field scene.

Efficient and Parallelizable PConv as an Alternative to Conventional Convolution: The introduction of multiscale feature layers is bound to increase the computational load of the model, slowing down its inference speed. Current research indicates that frequent memory access by operators is the primary cause of low FLOPS. To enhance the inference speed of the model as much as possible, we have employed a PConv that simultaneously reduces memory access time and computational redundancy, replacing conventional convolutions in the model. The working principle of PConv involves utilizing the first or last consecutive channel for continuous or regular memory access as a representative for the entire feature map, while the remaining channels remain unchanged [37]. As a result, the FLOPs of PConv are only:

4 $$h\times w\times {k}^{2}\times {c}_{p}^{2}.$$

With a typical partial ratio $r=\frac{{c}_{p}}{c}=\frac{1}{4}$, the FLOPs of a PConv is only $\frac{1}{16}$ of a regular Conv. Besides, PConv has a smaller amount of memory access, i.e.,

5 $$h\times w\times 2{c}_{p}+{k}^{2}\times {c}_{p}^{2}\approx h\times w\times 2{c}_{p}$$

which is only $\frac{1}{4}$ of a regular Conv for $r=\frac{1}{4}$.

### Model training and evaluation metrics

Parameter Configuration: To ensure the fairness of the experiments, identical initial training parameters are set for each group. Taking into account physical memory constraints and learning efficiency, the number of training images per batch is set to 4, and the maximum iteration count is set to 500. During training, the model employs the Stochastic Gradient Descent (SGD) [38] optimizer, and the learning rate ($lr$) decay strategy [39] can be described as follows:

6 $$lr=base\_lr\cdot {\left(1-\frac{iter\_num}{\text{max}\_iterations}\right)}^{p}$$

Here, $base\_lr$ represents the base learning rate, max_iterations is the maximum iteration count, $iter\_num$ is the iteration index, and p is the polynomial decay exponent. In this study, the base learning rate is set to 0.001, momentum is set to 0.9, weight decay is set to 1e−4, and the lower limit for learning rate updates is 0. These settings are consistently applied across all model training sessions.

This study employs the Cross-Entropy Loss function [39] to quantify the distance between the predicted probability distribution of pixel categories and the true label category probability distribution during the training process. The specific calculation method is as follows:

7 $$Loss=\frac{1}{M}\sum_{i=1}^{M}\sum_{C=1}^{N}h({b}_{i})\text{log}({p}_{ic})$$

In the formula, $M$ represents the number of pixels; $N$ represents the number of categories; $i$ represents the current pixel; $C$ represents the current category; ${b}_{i}$ is the true label category for pixel $i$; $h$ is the probability distribution function in the range of 0 ~ 1, where it is 1 if ${b}_{i}=c$ and 0 otherwise; ${p}_{ic}$ is the predicted probability of pixel i belonging to category $c$, obtained through the Sigmoid function applied to the calculation of predicted category scores. Through the computation of the loss function during the iteration process, the model's training performance is evaluated. The weights are adjusted through backpropagation to gradually reduce the error represented by the loss value, aiming to achieve the training objectives.

Evaluation Metrics: To quantitatively analyze the model's performance, this study employs Average Precision (AP), precision, and recall to assess the effectiveness of the proposed RMS-DETR model. For precision and recall, there are three states after the test sample is predicted: TP stands for True Positive, which represents the number of weed samples correctly detected by the model. FP stands for False Positive, indicating the number of incorrectly predicted weed samples. FN stands for False Negative, referring to the number of undetected weed samples.

8 $$precision=\frac{TP}{TP+FP}$$

9 $$recall=\frac{TP}{TP+FN}$$

The recall rate and precision rate are based on the threshold value of 0.5.

The experimental platform configuration is shown in Table 2.

**Table 2:** Experimental environment

Operating system	Hardware environment			Software environment			Machine learning framework
	CPU	Hard drive capacity	GPU	Python	cuDNN	CUDA	
Windows 10	Intel(R) Core(TM) i7-9700 @3.0 GHz	64G	NVIDIA GeForce RTX 5000	3.7	8.5.0	11.7	pytorch

## Experimental results

In this section, we conducted multiple experiments to validate the performance and reliability of the proposed RMS-DETR model in rice field weed detection. Comprehensive analysis and discussion of the experimental results were performed.

### Visualization

**Fig. 6:**
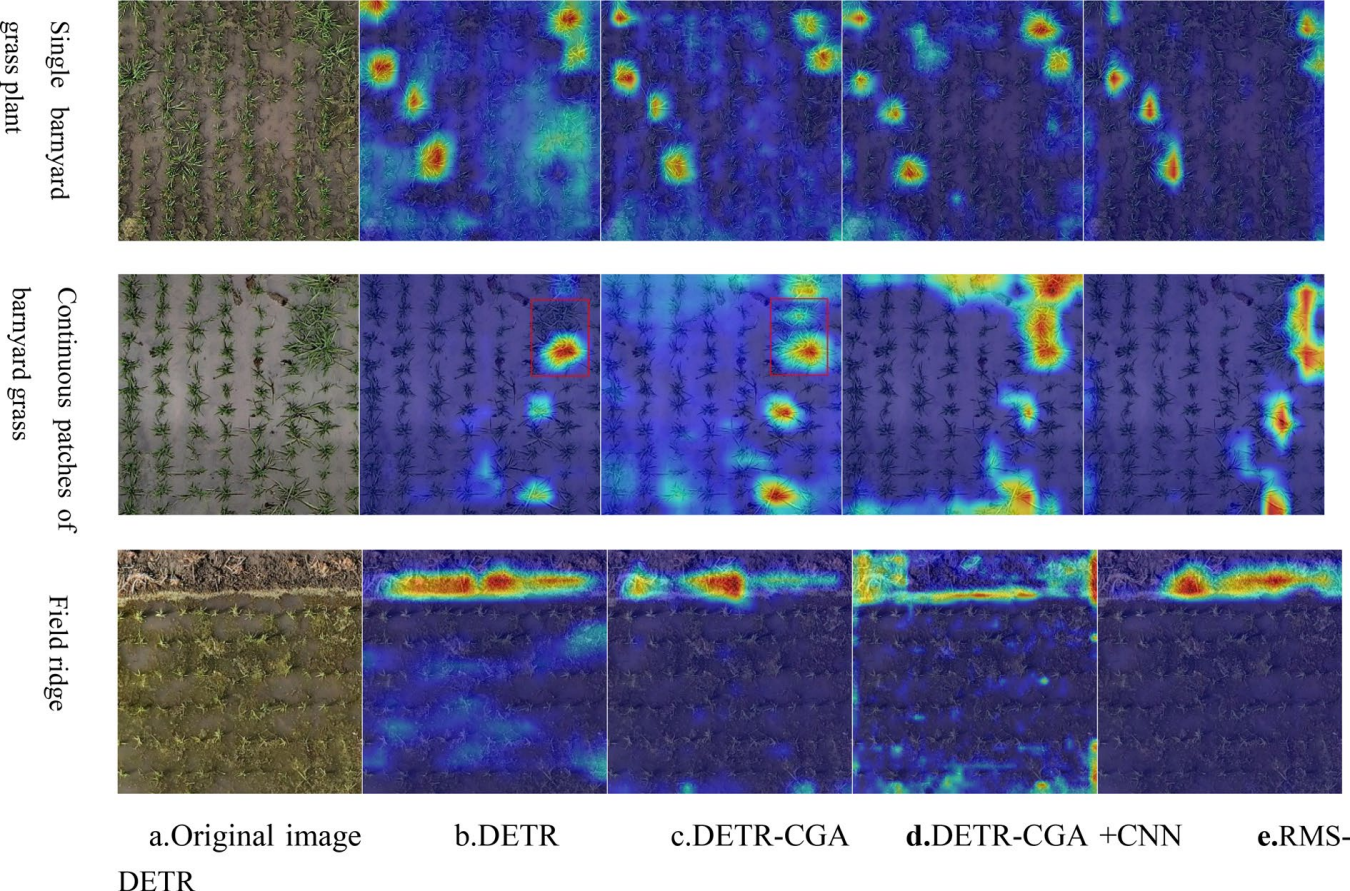
Visualization of target heatmaps under different improvement methods

To visually demonstrate the effectiveness of the proposed approach in improving the recognition performance of rice field weed images, this study introduced three successive improvements based on the original DETR model. The first improvement replaced the multi-head attention mechanism in DETR with CGA, resulting in DETR-CGA. The second improvement added multiscale feature layers to DETR-CGA and used CGA and CNN to extract high and low-level semantic features separately, yielding DETR-CGA + CNN. Finally, the RMS-DETR model was obtained by replacing conventional Conv with PConv on the basis of DETR-CGA + CNN. Subsequently, the attention maps for weed feature extraction were compared using Grad-CAM visualization technique between the original DETR, RMS-DETR, and the two intermediate variants. All attention maps are from the last encoding layer of the model's encoder. The results are shown in Fig. 6.

Observing Fig. 6c, b, it is evident that the DETR-CGA model, incorporating the CGAmodule, enhances attention to key feature regions when recognizing single barnyard grass plants and field ridges compared to the original DETR model. Although it expanded the attention scope on the features of contiguous weeds, the DETR-CGA model compensates for the missed detection issues present in the original DETR model, as illustrated by the red boxes in the figure. Observing Fig. 6e, d, it is evident that the RMS-DETR model, utilizing PConv, exhibits a pronounced focus in the attention distribution on the main feature regions of all target categories compared to the DETR-CGA + CNN model with conventional convolutions. The innovation of the RMS-DETR model lies in the effective fusion of global and local features. As depicted in Fig. 6e, when detecting single barnyard grass plants and continuous patches of barnyard grass, the RMS-DETR model primarily focuses on their growth positions between field ridges. The growth position of barnyard grass between field ridges is a typical local feature distinguishing barnyard grass from rice. When identifying field ridge categories, the RMS-DETR model emphasizes both the boundary parts of the field ridge and the presence of weeds on the ridge, ensuring comprehensive attention to both global and local features. This indicates that the RMS-DETR model, through the effective fusion of global and local features in the image, enhances the recognition ability of typical features in targets, thereby improving the detection performance of rice field weed by the model.

### Sensitivity analysis

To verify the contribution of the proposed improvement method to the model's performance, this study conducted ablation experiments based on a self-constructed rice field weed dataset. Starting with the framework of the original DETR base model, various improvement modules were progressively incorporated to create multiple model variants. The performance of each variant was then evaluated using the mAP50 metric, mAP50 indicator is based on the validation set results. Through ablation experiments, a quantitative analysis was conducted to assess the impact of each improvement method on the performance enhancement of the model in rice field weed detection tasks. The results are presented in Table 3.

**Table 3:** Recognition results of different improvement methods for rice field weeds

NO	Model	All	Single barnyard grass plant	Continuous patches of barnyard grass	Field ridge
1	DETR	0.764	0.647	0.77	0.875
2	DETR-CGA	0.772	0.687	0.81	0.818
3	DETR-CGA + CNN	0.784	0.73	0.782	0.839
4	RMS-DETR	0.792	0.686	0.816	0.873

1 → 2: In terms of overall accuracy, the DETR-CGA model has slightly improved in mAP50 metrics compared to the original DETR model, from 0.764 to 0.772. From various categories, compared to the DETR model, the DETR-CGA model has improved recognition accuracy by 4% in both single plant barnyard grass and Continuous patches of barnyard grass. This indicates that the CGA module enhances the model's ability to extract complex features, effectively improving the recognition accuracy of complex targets such as barnyard grass. However, we also observed a 6.5% decrease in model recognition accuracy when facing relatively regular and simple field ridge targets. The reason might be that the attention heads of the CGA module are overly concentrated on capturing crucial complex semantic information, leading to insufficient representation of simple low-level visual features and failing to provide effective support for simple targets.

2 → 3: The DETR-CGA + CNN model is built on the DETR-CGA model by introducing a multi-scale feature extraction module and effectively fusing the semantic information extracted from both Transformer and CNN structures. Its mAP50 overall score is improved from 0.772 to 0.784. This demonstrates that the effective fusion of global and local features is beneficial for enhancing target detection. For the single barnyard grass plant and field ridge categories, the recognition accuracy of the DETR-CGA + CNN model has been improved to varying degrees, especially the recognition accuracy for the single barnyard grass plant category, which has increased significantly. This shows that adding the multi-scale feature extraction module can improve the model's recognition accuracy for small target categories to some extent.

3 → 4: The RMS-DETR model, built on the DETR-CGA + CNN model, replaces the conventional convolutions with PConvs. This improvement effectively enhances the model's recognition capability, improving mAP50 overall score from 0.784 to 0.792. For large-area targets like continuous patches of barnyard grass and field ridges, the recognition accuracy of the RMS-DETR model has increased by 3.4% for both. However, for single barnyard grass plant, the recognition accuracy decreased by 4.4%. This suggests that the PConv structure may be more suitable for extracting features of large-area targets, while having limitations in extracting features of small-area targets.

### Analysis of other metrics

To more comprehensively analyze the impact of our proposed improvements on model performance, we utilize PR curves and AUC-PR values for performance evaluation.

**Fig. 7:**
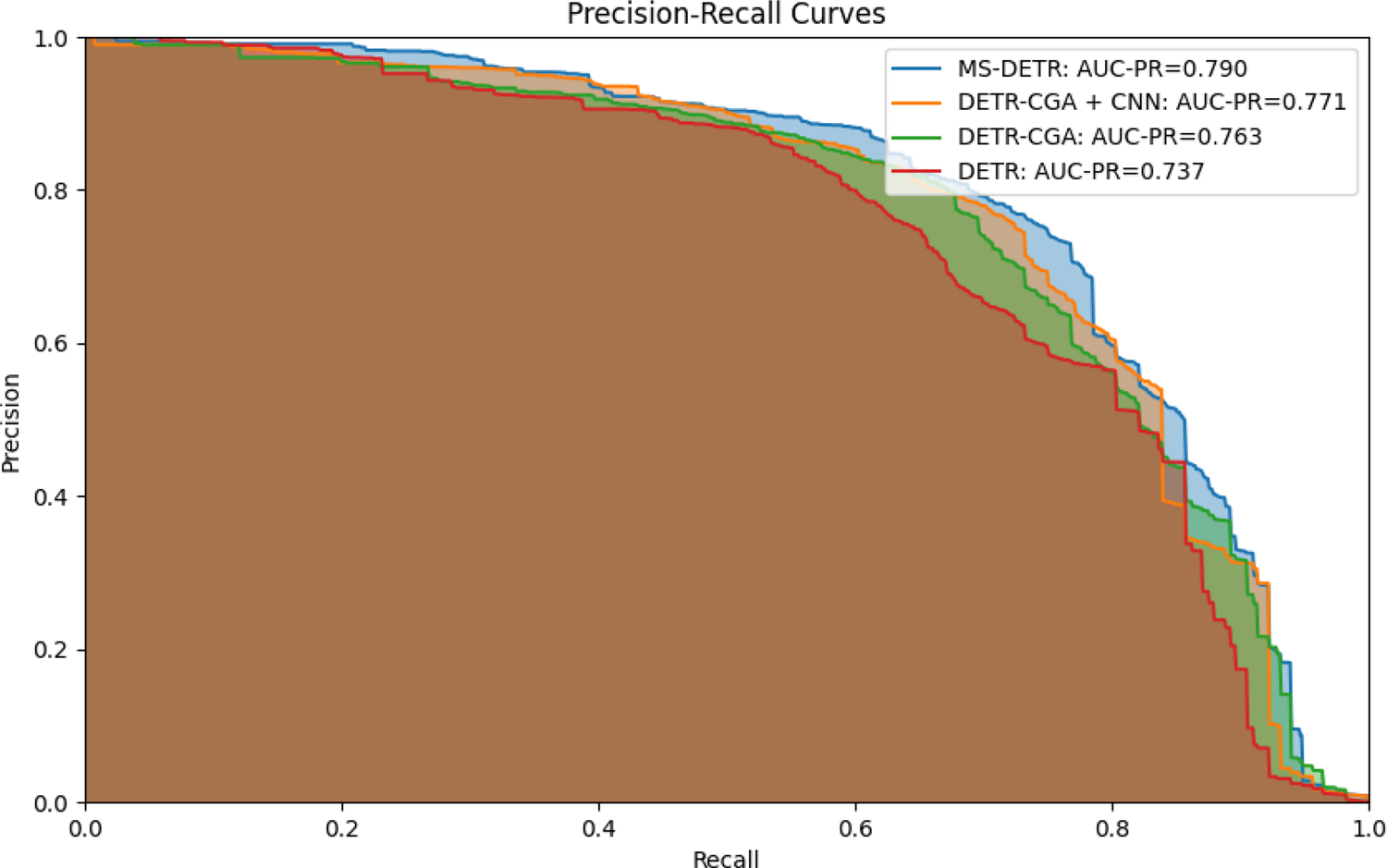
The precision-recall curves for models employing different improvement methods

Figure 7 depicts the precision-recall (PR) curves for models utilizing different improvement methods. The PR curve and the Area Under the Curve (AUC-PR) are commonly used metrics for evaluating model performance. The PR curve illustrates the relationship between precision and recall at various thresholds. It helps assess model performance across different thresholds. For model comparison, we can quantitatively evaluate by comparing the AUC-PR values, which range from 0 to 1. A PR curve closer to the upper-right corner of the plot corresponds to a higher AUC-PR value, indicating better model performance.

From Fig. 7, it can be observed that the PR curve of the RMS-DETR model is closest to the upper-right corner, indicating the best classification performance among the compared models. Additionally, its AUC-PR value reaches 0.79, surpassing other contrastive models, indicating that the RMS-DETR model has higher average recognition accuracy and better overall performance.RMS-DETRRMS-DETRRMS-DETR4.4 Validation of Enhanced Small Object Recognition Capability.

The detailed experiments in this section are to verify the enhanced effects of our proposed model on small target detection tasks. The model performance is evaluated by the mean Average Precision (AP) and mean Average Recall (AR) in different size ranges, where higher AP and AR values indicate better effects of the model in detecting targets within the corresponding size ranges. The AP and AR in Table 4 are obtained at IoU = 0.50:0.95. The subscripts are defined as follows: S represents small targets (area ≤ 32^2^), M represents medium targets (32^2^ < area ≤ 96^2^), L represents large targets (area > 96^2^), and area represents number of pixels [27].

**Table 4:** Recognition results of models with different improvement methods for different sizes of rice weeds

	RMS-DETR	DETR-CGA + CNN	DETR-CGA	DETR
AP-s	0.111	0.073	0.103	0.058
AP-m	0.153	0.139	0.136	0.118
AP-l	0.635	0.624	0.617	0.617
AR-s	0.266	0.443	0.364	0.191
AR-m	0.397	0.318	0.396	0.402
AR-l	0.807	0.798	0.814	0.788

The experimental results show that compared with the original DETR model, our proposed RMS-DETR model significantly improves the detection performance on large, medium and small targets. Among them, the gain on small target detection is the most significant, with AP and AR greatly improved by 91% and 39% respectively, outperforming all contrastive methods. The recognition of medium and large targets also has some improvement, with the AP of medium targets increased by 29%, and the AP and AR of large targets improved by 2.9% and 2.4% respectively. It should be noted that the AR of medium targets dropped slightly by 1.2%. The reason may be that the model optimization for small target detection resulted in less attention on medium targets. Since small target weeds are more densely distributed in weed scenes, the model optimization pays more attention to improving small target detection, which sacrifices the detection recall rate of medium-sized weed targets to some extent, leading to a slight 1.2% decline. Considering the small number and relatively easy detection of medium-sized weeds, such loss can be acceptable.

### Feasibility analysis of agricultural production

To assess the computational complexity of our proposed method, we conducted testing experiments on the collected rice weed dataset. To eliminate other influencing factors, we performed comparisons under the same experimental environment, where model parameters and GFLOPs were computed on a single NVIDIA RTX5000 GPU for input sizes of 640 × 640 pixels. Inference time was calculated as the average over 100 runs on test samples of 640 × 640 pixel images. The experimental results are presented in Table 5.

**Table 5:** Performance parameters of models with different improvement methods

Model	DETR	DETR-CGA	DETR-CGA + CNN	RMS-DETR
Parameters	38.6 MB	38.3 MB	42.5 MB	40.8 MB
Latency	0.00750 s ± 0.00145 s	0.00705 s ± 0.00095 s	0.00829 s ± 0.01191 s	0.00818 s ± 0.00105 s
FPS	133.3	141.8	120.6	122.2
mAP50	0.764	0.772	0.784	0.792

Compared with the original DETR model, the DETR-CGA model with the efficient CGA module reduced the model size by 0.3 MB. While reducing the number of parameters and model size, its accuracy was improved by 0.08, indicating that the CGA module provides different channel subsets of features as input to each head, which reduces model parameters while allowing each head to learn more unique features, thereby improving the model's recognition accuracy for rice field weeds. The DETR-CGA + CNN model introduced multi-scale feature layers later, with its number of parameters significantly increased by 9.7%, due to the additional parameters brought by the multi-scale feature layers. However, the model accuracy also increased by 0.012. On this basis, the efficient PConv was adopted to replace conventional convolutions in the RMS-DETR model. With no change in model structure, the number of model parameters decreased by 4%, FPS increased by 1.6, and model accuracy also improved by 0.08. Overall, compared with the original DETR model, our model has no significant advantages in terms of number of parameters and inference time. It completes weed recognition at a speed of 0.00818 s per image. Although not the fastest in inference, it achieved the best performance in recognition results. Our model strikes a good balance between recognition performance and computational efficiency, making it suitable for deployment on intelligent devices with limited computing power.

### Comparison on public datasets

To comprehensively evaluate the effectiveness of the proposed improvement methods, this study validates the original DETR model, various variant models, and the RMS-DETR model on the public DOTA dataset [27], The verification results are shown in Table 6. The DOTA dataset is a large-scale dataset for object detection in aerial images, containing 15 object categories such as small-vehicle, plane, and ship. It consists of 2,806 aerial images with a size of approximately 4,000 × 4,000 pixels, including 188,282 bounding boxes with various aspect ratios, orientations, and shapes. According to the COCO dataset's criteria for classifying small, medium, and large objects, the proportions of small, medium, and large objects in the DOTA dataset are 72.1%, 22.6%, and 5.3%, respectively, with a predominance of small objects, making it suitable for validating the proposed improvement methods. The mAP50 metric is adopted to evaluate the model's performance.

**Table 6:** Precision results of different improved models on the DOTA dataset

	DETR	DETR-CGA	DETR-CGA + CNN	RMS-DETR
mAP50	0.815	0.837	0.839	0.851
Small-vehicle	0.821	0.858	0.86	0.821
Large-vehicle	0.842	0.887	0.891	0.888
Plane	0.957	0.947	0.95	0.955
Storage-tank	0.9	0.925	0.916	0.919
Ship	0.837	0.87	0.848	0.859
Harbor	0.913	0.934	0.936	0.928
Ground-track-field	0.732	0.774	0.792	0.78
Soccer-ball-field	0.887	0.914	0.953	0.889
Tennis-court	0.979	0.993	0.972	0.995
Swimming-pool	0.852	0.943	0.932	0.955
Baseball-diamond	0.778	0.772	0.791	0.877
Roundabout	0.775	0.796	0.818	0.862
Basketball-court	0.822	0.839	0.718	0.841
Bridge	0.629	0.613	0.706	0.698
Helicopter	0.496	0.496	0.496	0.495

Specifically, replacing the traditional multi-head attention mechanism with CGA in the DETR-CGA model compared to the DETR model resulted in an overall accuracy improvement of 2.7%. In 11 categories such as swimming-pool, ground-track-field, and large-vehicle, the detection accuracy of the DETR-CGA model improved, with the swimming-pool category showing a significant increase of 10.7%. This result indicates that the CGA module has stronger feature capturing capabilities compared to the traditional attention mechanism in the object detection task on the DOTA dataset. Furthermore, after incorporating the CNN branch into the DETR-CGA model, the overall accuracy increased by 0.24%. The improved DETR-CGA + CNN model showed accuracy improvements across 10 categories, demonstrating that the CNN branch provides more diverse and rich feature representations, complementing the Transformer branch to provide comprehensive visual information and enhance detection performance. Additionally, the RMS-DETR model using partial convolution (PConv) showed an accuracy improvement in 8 categories compared to the DETR-CGA + CNN model using traditional convolution, while accuracy decreased in 7 categories. However, the overall accuracy increased by 1.4%. This indicates that PConv has a positive impact on model performance in the object detection task on the DOTA dataset.

Through experimental comparative analysis, the RMS-DETR model achieved improved detection accuracy in all categories except for small-vehicle, plane, and helicopter, compared to the original DETR model. The average precision increased by 4.4%. The DOTA dataset has a high proportion of small target instances, accounting for 72.1%, which poses higher demands on object detection models. Therefore, the performance improvement achieved by the RMS-DETR model on such datasets fully validates the effectiveness of the proposed model improvement scheme in this study.

### Comparison with other classic algorithms

**Fig. 8:**
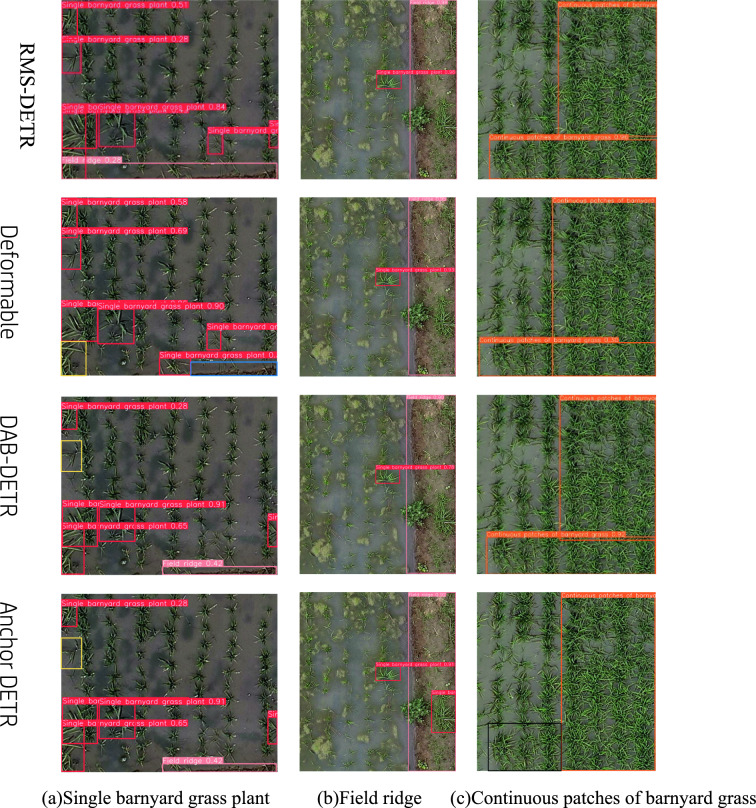
Recognition results of different models on rice weeds

In order to comprehensively evaluate the performance of the model on the rice weed detection task, we conducted comparative experiments on the rice weed dataset, comparing the RMS-DETR model with other classic DETR variants, including Deformable DETR [14], Anchor DETR [40], and DAB-DETR [41]. The experimental results are presented in Table 7 and Fig. 8.

Since Deformable DETR first introduced multi-scale features on the DETR basis, effectively improving the detection performance, and Anchor DETR and DAB-DETR are improved on the Deformable DETR model, we chose the above models for comparison. As shown in Table 7, among multiple DETR variants, RMS-DETR achieved the highest mAP50 value of 0.792, displaying the optimal recognition performance. In terms of the number of parameters, RMS-DETR used 40.8 M parameters, only higher than the smallest Anchor DETR (36.8 M). Considering the highest recognition accuracy of RMS-DETR, this means its parameter utilization efficiency is high. However, the computational complexity (GFLOPs) of RMS-DETR reached 187G, the largest among all compared models. Taking into account both recognition accuracy and parameter utilization efficiency, RMS-DETR achieved the best balance between the two, obtaining the highest recognition performance metrics, while keeping the number of parameters and computational complexity within a reasonable range.

**Table 7:** Detection performance of different DETR variant models

Model	RMS-DETR	Deformable DETR	Anchor DETR	DAB-DETR
mAP50	0.792	0.775	0.755	0.773
Parameters	40.8 M	41 M	36.8 M	44 M
GFLOPs	187G	86G	151G	94G

As shown in Fig. 8, our proposed RMS-DETR model performs the best in recognizing smaller single barnyard grass plant targets, accurately identifying all weed instances and field ridges in the image, while the Deformable DETR and DAB-DETR models failed to detect the field ridge in the bottom right corner (as shown in the blue box in Fig. 8a), and missed detecting some weeds (as shown in the yellow box in Fig. 8a). The reasons may be: first, Deformable DETR does not distinguish between feature layers at different scales. The independent Deformable Attention modules on low semantic feature layers cannot effectively capture detailed features like CNNs. They do not fully exploit the key localization information that low semantic layers provide for small targets; Second, the multi-scale feature extraction and fusion process of simple “stacking-summing” is too singular to model the rich interactions between features, which limits the effectiveness of multi-scale information representation and integration of the model. Although the Anchor DETR model detected the field ridges, it also missed some weed targets (as shown in the yellow box in Fig. 8a).

For larger field ridge targets, all models can identify them relatively well. However, the Anchor DETR model incorrectly identified the barnyard grass on the field ridges, which should not have been annotated during the data annotation process. Therefore, there was no dataset with barnyard grass on field ridges in the training data, resulting in a kind of false positive detection. For recognizing continuous patches of barnyard grass, Anchor DETR failed to detect the continuous patches of barnyard grass in the bottom left corner (as shown in the black box in Fig. 8c), while other models basically detected the area of continuous weed patches, but with some differences in the positioning of detection boxes. The RMS-DETR model left a small unlabeled area in recognizing continuous patches of barnyard grass, while the detection boxes of Deformable DETR and DAB-DETR models have some overlap, especially the two boxes in the Deformable DETR model with the largest overlap area. The possible reasons for Anchor DETR missing a patch of weed target (as shown in the black box in Fig. 8c) are: (1) The concept of "dense weeds" itself is relatively subjective, and different people have different understandings and criteria regarding weed density. Even for the same person, the understanding of "dense" may change when annotating data at different times, resulting in inconsistent labels in the training data. (2) The current training data volume is relatively small, and the samples of various weed density scenarios are not comprehensive enough. This limits the model's ability to learn the concept of “dense weeds”.

## Discussion

Due to the high similarity in morphology between barnyard grass and rice plants, and the fact that barnyard grass are small objects in UAV remote sensing imagery, accurate identification of barnyard grass in rice fields based on UAV remote sensing is challenging. To address this problem, this study proposes targeted improvement measures and develops a rice field barnyard grass object detection model to handle barnyard grass detection tasks in complex real-world scenarios.

In order to improve the recognition accuracy of barnyard grass in remote sensing imagery, we proposed the RMS-DETR model, which introduces multi-scale feature layers on the basis of DETR. We designed the different feature layers differently. The high-level semantic feature layer adopts Transformer structure to emphasize the extraction of context relationship information between barnyard grass and rice plants. The low-level semantic feature layer uses CNN structure to extract barnyard grass detail features. This is because high-level semantic feature layers usually contain more abstract and semantic information. The self-attention mechanism in Transformers allows each input position to associate with all other positions, unlike CNN networks which are limited by fixed window sizes. This fully-connected mechanism enables the model to build relationships between any two pixels in the image, thereby better extracting global feature information. Low-level semantic feature layers usually contain more detailed information. The process of convolving the convolution kernels with the feature layer element-by-element in CNN networks is essentially weighted aggregation of features, which can effectively capture local features in the feature layer.

When using Transformer structure to extract context information of rice field weeds, we introduced the CGA module to replace the traditional multi-head attention mechanism in Transformer structure. Since the CGA module splits the input features into multiple channel subsets and takes these channel subsets as the inputs to different self-attention heads separately, it avoids repetitive encoding of the same information by different heads and reduces computational redundancy. Meanwhile, different heads extracting features from their own channel subsets help the model learn more diverse representations of the input features. Experimental results show that this improvement increased the detection accuracy (mAP50) by 1%, reduced the model size from 38.6 to 38.3 M, and shortened the inference time from 0.0075 to 0.00705 s.

When using CNN to extract barnyard grass detail features, we apply atrous convolutions with different dilation rates on the same semantic feature layer to achieve multi-scale observation of the feature layer, thereby enabling the model to capture small barnyard grass features. Experimental results show that this improvement increased the barnyard grass recognition accuracy by 1.6%. This is mainly attributed to the enlarged receptive field of convolution kernels by introducing dilation rates in atrous convolution, which can capture richer features of barnyard grass objects and surrounding background regions. However, the introduction of this multi-branch structure leads to increased computational burden and slower inference speed. The model size increased from 38.3 to 42.5 M, and the detection time increased from 0.00705 to 0.00829 s.

In order to maximize the model's inference speed, we extensively adopted the efficient parallelizable PConv in the model to replace conventional convolutions. PConv treats the first or last consecutive channel subset of the feature map as the representative of the entire feature map, performs spatial feature extraction on it using Conv, while keeping the remaining channels unchanged. This strategy of focusing only on key channels significantly improves computational efficiency and reduces channel redundancy. Experimental results show that the use of PConv modules not only reduced model parameters from 42.5 to 40.8 M, but also improved average inference time by 1.3%. More importantly, the barnyard grass detection accuracy also increased from 0.784 to 0.792.

Although the RMS-DETR model performs well on our self-built rice field weed dataset, the improvement in recognition accuracy comes at the cost of increased model parameters. Moreover, there are still many factors that have not been evaluated in this study.RMS-DETR First, our training set was collected from a single experimental field, without considering the effects of different farm management measures on dominant weed species. Second, changes in lighting conditions may affect image features, while the current dataset does not cover variations under different weather conditions. These two limitations may affect the model's generalization ability in other environments. To mitigate the above effects, in future research, we will collect rice field weed datasets across more regions and time spans, to include samples under varying lighting conditions and with different weed species, so as to expand the applicability of the RMS-DETR model.

## Conclusion

The main conclusions of this study, which proposes a rice field weed detection method for UAV remote sensing, are summarized as follows:

1. By introducing multi-scale feature layers in the DETR model and differentiating their designs, the detection performance of the DETR model can be effectively improved, especially for detecting small targets. Compared with the original DETR model, the overall detection accuracy of our proposed RMS-DETR model is improved by 3.6%, and the detection accuracy for small targets is increased substantially by 91%.
2. Incorporating the CGA module into the DETR model to replace the traditional multi-head attention mechanism can effectively reduce model computation while improving detection accuracy. The model size is reduced by 0.3 M and the overall detection accuracy is improved by 1%.
3. Extensively using PConv in the model can effectively decrease model computation. The model size is reduced by 1.7 M.

## Data Availability

No datasets were generated or analysed during the current study
